# Low Trap Density Para-F Substituted 2D PEA_2_PbX_4_ (X = Cl, Br, I) Single Crystals with Tunable Optoelectrical Properties and High Sensitive X-Ray Detector Performance

**DOI:** 10.34133/2022/9768019

**Published:** 2022-10-10

**Authors:** Jiayu Di, Haojin Li, Li Chen, Siyu Zhang, Yinhui Hu, Kai Sun, Bo Peng, Jie Su, Xue Zhao, Yuqi Fan, Zhenhua Lin, Yue Hao, Peng Gao, Kui Zhao, Jingjing Chang

**Affiliations:** ^1^State Key Discipline Laboratory of Wide Band Gap Semiconductor Technology, School of Microelectronics, Xidian University, 710071 Xi'an, China; ^2^Advanced Interdisciplinary Research Center for Flexible Electronics, Academy of Advanced Interdisciplinary Research, Xidian University, 710071 Xi'an, China; ^3^Key Laboratory of Applied Surface and Colloid Chemistry, National Ministry of Education, School of Materials Science and Engineering, Shaanxi Normal University, 710119 Xi'an, China; ^4^CAS Key Laboratory of Design and Assembly of Functional Nanostructures, Fujian Institute of Research on the Structure of Matter, Chinese Academy of Sciences, Fuzhou 350002, China

## Abstract

Exploring halogen engineering is of great significance for reducing the density of defect states in crystals of organic-inorganic hybrid perovskites and hence improving the crystal quality. Herein, high-quality single crystals of PEA_2_PbX_4_ (X = Cl, Br, I) and their para-F (*p*-F) substitution analogs are prepared using the facile solution method to study the effects of both *p*-F substitution and halogen anion engineering. After *p*-F substitution, the triclinic PEA_2_PbX_4_ (X = Cl, Br) and cubic PEA_2_PbX_4_ (X = I) crystals unifies to monoclinic crystal structure for *p*-F-PEA_2_PbX_4_ (X = Cl, Br, I) crystals. The *p*-F substitution and halogen engineering, together with crystal structure variation, enable the tunability of optoelectrical properties. Experimentally, after the *p*-F substitution, the energy levels are lowered with increased Fermi levels, and the bandgaps of *p*-F-PEA_2_PbX_4_ (X = Cl, Br, I) are slightly reduced. Benefitting from the enhancement of the charge transfer and the reduced trap density by *p*-F substitution and halogen anion engineering, the average carrier lifetime of the *p*-F-PEA_2_PbX_4_ is obviously reduced. Compared with PEA_2_PbI_4_, the X-ray detector based on *p*-F-PEA_2_PbI_4_ perovskite single-crystal has a higher sensitivity of 119.79 *μ*C Gy_air_^−1^·cm^−2^. Moreover, the X-ray detector based on *p*-F-PEA_2_PbI_4_ single crystals exhibits higher radiation stability under high-dose X-ray irradiation, implying long-term operando stability.

## 1. Introduction

X-ray detectors, as the core components of human medical diagnostic machines and security inspection machines, can convert X-rays into identifiable electrical signals [1–3]. Compared with scintillators, direct-type X-ray detectors have better energy resolution [4]. Generally, the performance of the device is closely related to the average atomic number, carrier mobility, and charge carrier lifetime product of semiconductors [5, 6]. At present, the largest market share of direct-type X-ray detectors is *α*-Se, but its low atomic numbers became a limitation of its further development [7]. In addition, the common semiconductor materials used for X-ray detection have corresponding defects, including the following: GaAs, which has low resistivity and is challenging to prepare single crystals [8], and CdZnTe, which has high crystal preparation cost [9, 10]. The excellent optoelectronic properties and elements adjustability of perovskite materials make them stand out among direct X-ray detectors and have attracted extensive attention from researchers [11–16].

In order to obtain high-performance X-ray detectors, it is crucial to realize perovskite single crystals with low defect state density [17, 18]. During the past few years, perovskite single crystals based on low-cost solution methods have considerably developed [19–22]. Although perovskite single crystals have significantly reduced the effect of defect states at grain boundaries compared to their thin-film counterparts, the presence of defects in crystals cannot be ignored. Therefore, great efforts were made to reduce the impact of defects on the surface of three-dimensional (3D) perovskite single crystals on device performance. Previously, Huang et al. pioneered the treatment of MAPbBr_3_ by UV-ozone post-treatment to improve the carrier extraction efficiency [23]. After that, Dong et al. used MAI to passivate the surface of MAPbI_3_ single crystal to improve the performance of X-ray detectors and solar cells, respectively [24, 25]. These approaches aim to reduce the formation of charge traps caused by the more easily generated ion migration in 3D perovskites [26–29]. However, it is still urgently desired to explore low-defect state perovskite materials that can suppress ion migration by themselves under electric fields.

The emergence of two-dimensional (2D) perovskites with outstanding properties has ameliorated the above problems [30–32]. The larger organic cations in 2D perovskites expand the crystal lattice and form layered structures, which can block the migration of halide ions, thus endowing them with better stability [33]. Although the 2D perovskite single crystal has been widely used to improve the overall hydrophobicity and humidity stability, it remains challenging to realize high-performance perovskite-based X-ray detectors with low density of defect states. Recently, the study on *p*-F-PEA_2_PbI_4_ composed of F substituted at the para-position in PEA found that the dipole interaction introduced by F substitution enhances the *π*-*π* stacking between the benzene rings resulting in better-aligned stacking of perovskite sheets with reduced trap density [11, 34–38]. You et al. used first-principle calculations to prove that *p*-F-PEA_2_PbI_4_ has a smaller formation energy and less crystal disorder, which is conducive to the ordered growth of crystals and is more conducive to improving the performance of the device [39]. Besides, the change of the halogen element X (X = Cl, Br, I) in the perovskite will directly affect the length and angle of the Pb-X bond, hydrogen bond strength, and structural stability of the perovskite single crystal [40, 41]. Generally, the introduction of the F atom could alter the charge distribution of PEA, affect the electron density of the N-H bond, and enhance the N-H∙∙∙X hydrogen bond due to the electronic inductive effect. In short, dipole interaction, hydrogen bond, and Pb-X bond are crucial for regulating crystal stacking, which is also the key to further improving the quality of 2D perovskite crystals [42]. To better reveal their structure-activity relationship and halogen engineering effects, we explored the growth, and properties of a series of 2D PEA_2_PbX_4_ (X = Cl, Br, I) perovskite single crystals and their F substitution analogs. Furthermore, corresponding X-ray detectors were fabricated to explore the improvement of device performance.

Herein, we designed and synthesized PEA_2_PbX_4_ and *p*-F substituted PEA_2_PbX_4_ (X = Cl, Br, I) single crystals by a simple solution method. The *p*-F substitution and halogen anion engineering are demonstrated to have significant effects on the crystal structure of the *p*-F substituted products noted as *p*-F-PEA_2_PbX_4_ (X = Cl, Br, I). After *p*-F substitution, either the triclinic PEA_2_PbX_4_ (X = Cl, Br) or the cubic PEA_2_PbI_4_ crystals changed into monoclinic crystal structure when acquiring *p*-F-PEA_2_PbX_4_ (X = Cl, Br, I) crystals. As the halogen anions change following the sequence, Cl^−^ ⟶ Br^−^ ⟶ I^−^, the bandgap of the PEA_2_PbX_4_ (X = Cl, Br, I) and *p*-F-PEA_2_PbX_4_ (X = Cl, Br, I) decreases consistently. The F substitution and halogen engineering directly change the *π*-*π* stacking of the organic cation layer, which not only affects the crystal structure, but also leads to the shift of the energy band structure. Furthermore, the larger dipole moment caused by F substitution could induce an electronic inductive effect to enhance the hydrogen bond between N-H and X atoms. The enhancement of electron cloud stacking and hydrogen bond strength also dramatically reduces the defect density of the single crystal, which is a critical factor for the modification of device performance. Most importantly, the transport anisotropic properties of 2D perovskite single crystals make it an obvious advantage in horizontal structure devices. Compared with PEA_2_PbI_4_, a planar X-ray detector based on *p*-F-PEA_2_PbI_4_ perovskite single crystal has a higher sensitivity of 119.79 *μ*C Gy_air_^−1^·cm^−2^ and a low detection limit of ~1 *μ*Gy_air_·s^−1^, which meets the needs of medical diagnosis. Moreover, the performance of the *p*-F-PEA_2_PbI_4_ single-crystal X-ray detector is more stable under high-dose X-ray irradiation, proving that the introduction of F significantly improves the crystal quality, which is very critical to satisfying the long-term working conditions of X-ray detectors.

## 2. Results

**Figure 1:**
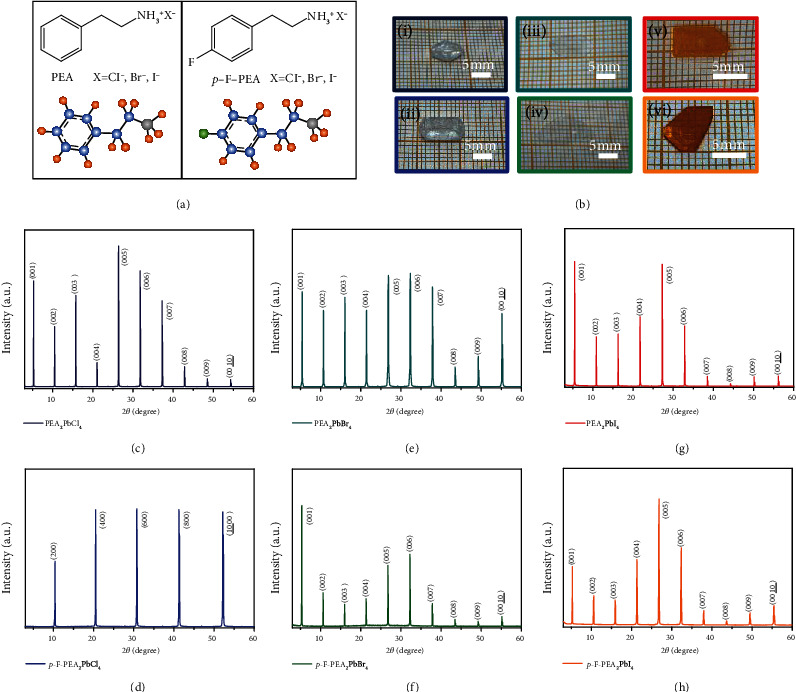
Photographs and XRD patterns of six materials. (a) The structural formula of PEAX and *p*-F-PEAX. (b) Photograph of (i) PEA_2_PbCl_4_, (ii) *p*-F-PEA_2_PbCl_4_, (iii) PEA_2_PbBr_4_, (iv) *p*-F-PEA_2_PbBr_4_, (v) PEA_2_PbI_4_, and (vi) *p*-F-PEA_2_PbI_4_ single crystals. XRD pattern of (c) PEA_2_PbCl_4_, (d) *p*-F-PEA_2_PbCl_4_, (e) PEA_2_PbBr_4_, (f) *p*-F-PEA_2_PbBr_4_, (g) PEA_2_PbI_4_, and (h) *p*-F-PEA_2_PbI_4_, respectively.

PEA_2_PbCl_4_ and PEA_2_PbBr_4_ single crystals are obtained by slow solution evaporation method (Figure S1a) [21, 43, 44]. The PEA_2_PbI_4_ single crystal was grown by slow cooling, the most common crystal growth method ever reported (Figure S1b) [11, 45]. The photos of the as-prepared single crystal are shown in (i, iii, and v) in Figure 1(b). Considering the large electronegativity of F and the negligible influence of para substitution on the symmetry of the benzene ring in the PEA^+^ cations, the effect of the *p*-F substitution on the properties of PEA_2_PbX_4_ (X = Cl, Br, I) was studied. We have prepared the PEA_2_PbX_4_ (X = Cl, Br, I) single crystals and the corresponding *p*-F substitution products noted as *p*-F-PEA_2_PbCl_4_, *p*-F-PEA_2_PbBr_4_, and *p*-F-PEA_2_PbI_4_, respectively. The *p*-F-PEA_2_PbCl_4_ and *p*-F-PEA_2_PbBr_4_ single crystals are grown by the same slow solution evaporation method with the PEA_2_PbCl_4_ and PEA_2_PbBr_4_. Similarly, *p*-F-PEA_2_PbI_4_ single crystals are grown by the same reducing temperature method as its analog. The horizontal areas of the as-prepared 2D single crystals in our work can reach centimeter-scale, as shown in Figure 1. We used an optical microscope to observe the *p*-F-PEA_2_PbBr_4_ single crystals during the growth process and re-dissolved the flocculent solution to ensure the single-crystal quality (Figure S2). Notably, the *p*-F-PEA_2_PbCl_4_ single-crystal must be preserved in the glove box or encapsulated to avoid crystal decomposition (Figure S3).

XRD measurements were performed on the top surface (growth trend surface) of the single crystals in Figure 1(b). As shown in Figures 1(c)–1(h), these single crystals have high-intensity XRD diffraction patterns, confirming that the grown crystals have higher phase purity. The preferential growth surface of both PEA_2_PbX_4_ (X = Cl, Br, I) and *p*-F-PEA_2_PbX_4_ (X = Br, I) is the (001) plane, indicating that the single crystal grows in solution preferentially along with the organic layer or the inorganic layer plane, which leads to the best development of the (001) plane. However, *p*-F-PEA_2_PbCl_4_ showed a different preferential growth surface of the (100) plane. As shown in Table S1, PEA_2_PbI_4_ belongs to a cubic system, and the PEA_2_PbX_4_ (X = Cl, Br) belongs to a typical triclinic system (a ≠ b ≠ c, *α* ≠ *β* ≠ *γ*), with the space group P-1. Interestingly, all of their *p*-F substitution, *p*-F-PEA_2_PbX_4_ (X = Cl, Br, I), belongs to the monoclinic system (a ≠ b ≠ c, *α* = *γ* =90°, and *β* ≠90°).

**Figure 2:**
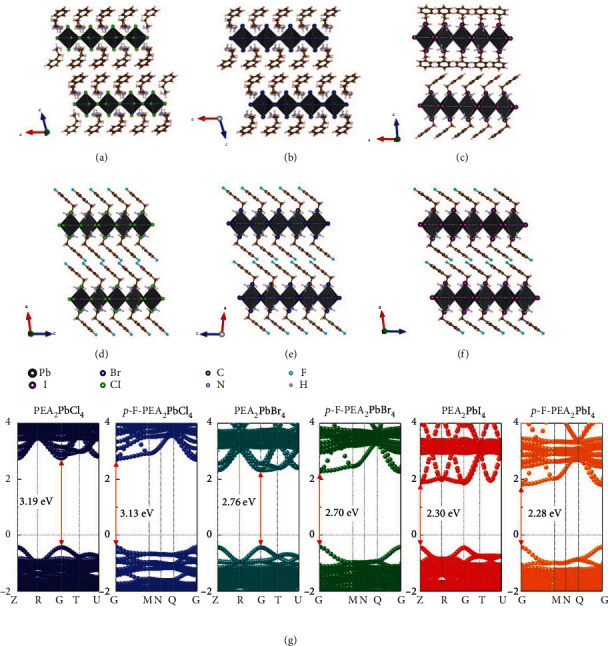
The crystal structures of (a) PEA_2_PbCl_4_, (b) PEA_2_PbBr_4_, (c)PEA_2_PbI_4_, (d) *p*-F-PEA_2_PbCl_4_, (e) *p*-F-PEA_2_PbBr_4_, and (f) *p*-F-PEA_2_PbI_4_. (g) Band structures of PEA_2_PbX_4_ and *p*-F-PEA_2_PbX_4_ (X = Cl, Br, I).

In order to further explore the *p*-F substitution organic cation and halide anion changes on the properties of single crystals, the crystal structures and orientation of PEA_2_PbX_4_ and *p*-F-PEA_2_PbX_4_ (X = Cl, Br, I) are shown in Figures 2(a)–2(f). The corner-shared [PbX_6_]_2_^−^ octahedra stacked in the [001] direction forms inorganic layers in 2D RP (PEA)_2_PbX_4_, each of which is neutralized and separated by PEA^+^ organic cations. After the *p*-F substitution, the corner-sharing structure, the polyoctahedral stacking ways in the inorganic layer, and the filling position of *p*-F-PEA^+^ molecules remain unchanged in the *p*-F-PEA_2_PbX_4_ counterparts (Figures 2(d)–2(f)). The large electronegativity of F in the organic layer changes the *π*-*π* stacking of *p*-F-PEA^+^ molecules from edge-to-edge stacking (also known as T-type stacking) to offset stacking (F-type stacking, the other type is edge-to-edge stacking) [46, 47]. The enhancement of intermolecular packing eventually caused the crystal system to change from triclinic and cubic to monoclinic. Furthermore, Perdew-Burke-Ernzerhof functional within the generalized gradient approximation (GGA-PBE) was used to research the bandgaps change of the six crystals, as shown in Figure 2(g). The bandgap of the PEA_2_PbX_4_ (X = Cl, Br, I) decreases as the halogen anions change following the sequence, Cl^−^ ⟶ Br^−^ ⟶ I^−^, while the radius of the halide increases. After F substitution, the bandgap of *p*-F-PEA_2_PbCl_4_ was smaller than PEA_2_PbCl_4_, which is the same situation with the PEA_2_PbX_4_ (X = Br, I). The band variation of crystals was attributed to the change of the *π*-*π* stacking as well as the electronic interaction after the *p*-F substitution.

**Figure 3:**
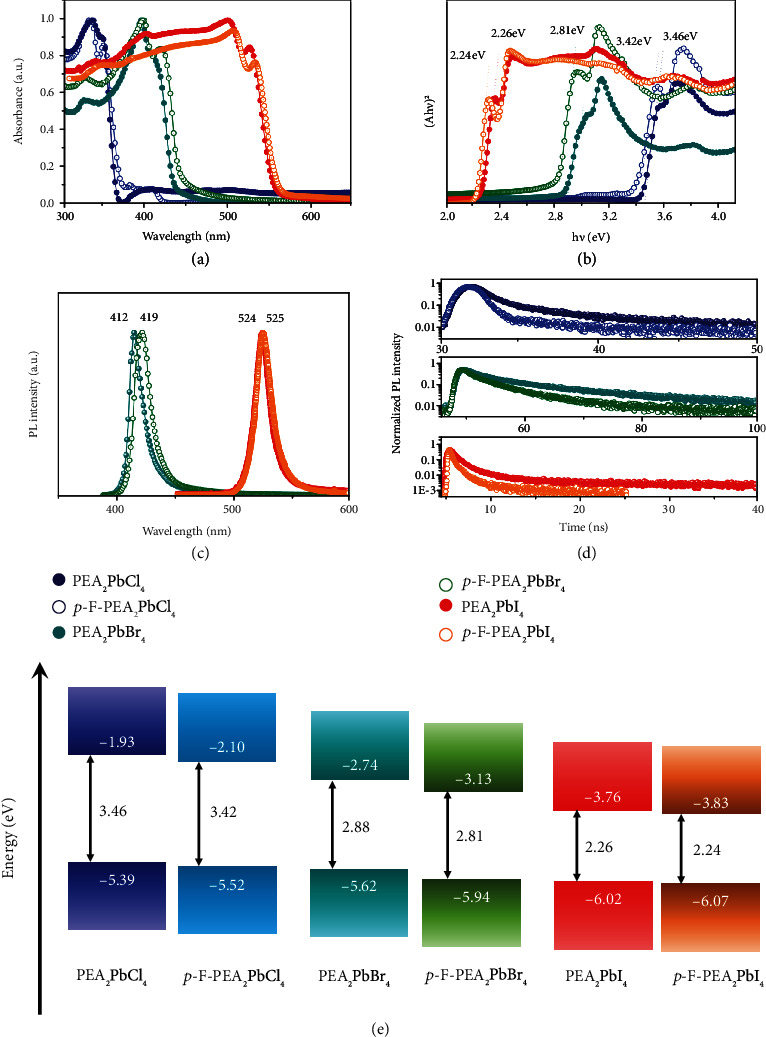
Characterization results of six materials. (a) UV-vis absorption spectra of PEA_2_PbX_4_ and *p*-F-PEA_2_PbX_4_ (X = Cl, Br, I). (b) Tauc plot for PEA_2_PbX_4_ and *p*-F-PEA_2_PbX_4_ (X = Cl, Br, I). (c) PL spectrum of PEA_2_PbX_4_ and *p*-F-PEA_2_PbX_4_ (X = Br, I). (d) TR-PL spectrum of the PEA_2_PbX_4_ and *p*-F-PEA_2_PbX_4_ (X = Cl, Br, I). (e) The energy level diagram of the PEA_2_PbX_4_ and *p*-F-PEA_2_PbX_4_ (X = Cl, Br, I).

As shown in Figure 3(a), the UV-Vis spectra of PEA_2_PbX_4_ and *p*-F-PEA_2_PbX_4_ (X = Cl, Br, I) indicated that the F substitution on organic cation affects the absorption spectra of the perovskites. After F substitution, the absorption edges of PEA_2_PbCl_4_, PEA_2_PbBr_4_, and PEA_2_PbI_4_ are red-shifted, and the corresponding bandgap values are slightly decreased from 3.46 eV to 3.42 eV, from 2.88 eV to 2.81 eV, and from 2.26 eV to 2.24 eV, respectively. The bandgap of these materials extracted from UV-vis spectra by the Tauc plot method is shown in Figure 3(b). The change of the absorption edge originates from the change of the *π*-*π* stacking way of the benzene ring in the organic molecular layer accompanied by the crystal structure variation (Figures 2(a)–2(f)). We then investigated the photoluminescence (PL) of the six crystals under ambient conditions (Figure 3(c)). The PL peak positions of the PEA_2_PbX_4_ (X = Cl, Br, I) show a red-shift after F substitution, with the PL peak position shifting from 521 nm to 528 nm for PEA_2_PbCl_4_, from 412 nm to 419 nm for PEA_2_PbBr_4_, and from 524 nm to 525 nm for PEA_2_PbI_4_, respectively. In particular, PEA_2_PbCl_4_ and *p*-F-PEA_2_PbCl_4_ exhibit good white-light fluorescence emission characteristics, as shown in Figure S5. All of the PL changes for the six PEA_2_PbX_4_ (X = Cl, Br, I) crystals are in accordance with their bandgaps. The wavelength of the excitation laser, PL peak, and full width at half maxima of the materials are listed in Table S2 to provide a deep insight into the luminescence properties. To ascertain the structure-PL properties relationship, we tested the time-resolved PL (TR-PL) spectroscopy of PEA_2_PbX_4_ and *p*-F-PEA_2_PbX_4_ single crystals. The applied excitation laser wavelength and the corresponding TR-PL results are shown in Figure 3(d). The TR-PL curves of these materials follow the double exponential fitting, and the average carrier lifetime (*t*_*av*_) values are obtained by double exponential fitting, as summarized in Table S3. It can be clearly seen that the *t*_*av*_ of both PEA_2_PbX_4_ and *p*-F-PEA_2_PbX_4_ single crystals are maintained at the ~ ns level. Moreover, the *t*_*av*_ values of *p*-F-PEA_2_PbX_4_ (X = Cl, Br, I) decrease after the *p*-F substitution, indicating thatintroducing F ions enhances the charge transfer inside the crystals [48]. The Bader charge transfers between supramolecular and Pb-I octahedron and between supramolecular and supramolecular of PEA_2_PbX_4_ and *p*-F-PEA_2_PbX_4_ (X = Cl, Br, I) are listed in Table S4. The charge transfer of the *p*-F-PEA_2_PbX_4_ is faster than PEA_2_PbX_4_ (X = Cl, Br, I), benefitting the X-ray detection.

Ultraviolet photoelectron spectroscopy (UPS) was measured for investigating the energy levels of (PEA)_2_PbX_4_ and *p*-F-PEA_2_PbX_4_ single crystals (Figure S6). Figure 3(e) shows that the energy band changes of materials before and after F substitution. The positions of conduction band minimum (CBM) and the valence band maximum (VBM) are slightly reduced after F substitution. The bandgap changes of the six materials are consistent with the material bandgap changes calculated by DFT. The voltage-current (*I-V*) curves of the six single crystals were tested to evaluate the quality of single crystals. In general, the trap state of the material is obtained based on the space-charge-limited current (SCLC) based on the Equation (1). When the applied voltage gradually increases, the device current enters the trap-filled limited (TFL) region from the ohmic region, and the inflection point of the *I–V* curve is the trap- filled limit voltage (*V*_TFL_), which is used to calculate the trap density (*n*_trap_) of the material.

(1) $$\begin{array}{c} n_{\text{trap}}=\frac{2{\varepsilon \varepsilon }_{0}V_{TFL}}{{qL}^{2}}, \end{array}$$

where *ε* is the relative dielectric constant, *ε*_0_ is the dielectric constant in a vacuum, *q* is the unit charge, and *L* is the thickness of the device. *I-V* tests were performed on the six single crystals based on hole-only devices (and electron-only devices (Figures S7 and S8), and their electron trap densities and hole trap densities are summarized in Table S5. All the 2D PEA_2_PbX_4_ and *p*-F-PEA_2_PbX_4_ perovskite single crystals show significantly fewer trap states (10^9^~10^11^ cm^−3^) than the typical semiconductor materials [18], such as Si (*N*_traps−si_ = 10^13^ ~ 10^14^ cm^−3^) [49], CIGS (*N*_traps−CIGS_ ~ 10^13^ cm^−3^) [50], and CdTe (*N*_traps−CdTe_ = 10^11^ ~ 10^13^ cm^−3^) [51]. It is worth noting that the defect state density of *p*-F-PEA_2_PbX_4_ material is one order of magnitude smaller than that of PEA_2_PbX_4_ and the crystal quality after F substitution is improved. The formation energies of the halide ion vacancy defect of PEA_2_PbX_4_ and *p*-F-PEA_2_PbX_4_ are shown in Figure S9, and the corresponding DFT calculated values are listed in Table S6. The results show that the defect formation energies are increased after F substitution, indicating that the material with F substitution has better structural stability. It means that the introduction of a large dipole effect after F substitution plays a positive role in reducing the defect states density of single-crystal materials. Moreover, as the electronegativity of halide ions increases (from I^−^ to Cl^−^), the defect formation energy is greatly improved, indicating that the overall stability of the material is improved. Both theoretical and experimental results show that the crystal quality has been greatly improved through the enhancement and synergy of the interlayer *π*-*π* stacking, dipole interactions, as well as halide change-induced self-assembly.

**Figure 4:**
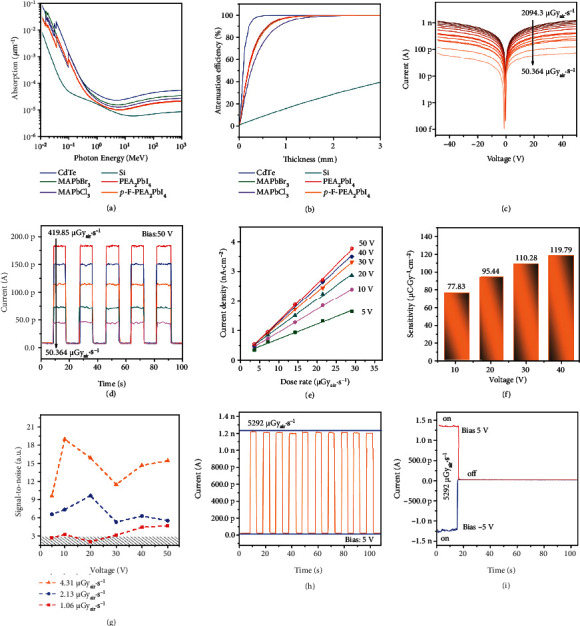
X-ray detector test results for *p*-F-PEA_2_PbI_4_. (a) Absorption coefficients of *p*-F-PEA_2_PbI_4_, PEA_2_PbI_4_, CdTe, MAPbBr_3_, MAPbCl_3_, and silicon versus different X-ray photons energy. (b) Attenuation efficiency of *p*-F-PEA_2_PbI_4_, PEA_2_PbI_4_, CdTe, MAPbBr_3_, MAPbCl_3_, and silicon for 40 keV X-ray photons versus thickness. (c) *I–V* curves of the *p*-F-PEA_2_PbI_4_ single-crystal X-ray detector measured with dose rate from 50.364 to 2094.3 *μ*Gy_air_·s^−1^. (d) ON/OFF current response of *p*-F-PEA_2_PbI_4_ single-crystal devices at 50 V bias voltage. The dose rate is from 50.364 to 419.85 *μ*Gy_air_·s^−1^. (e) X-ray generated current density versus dose rate under different bias voltages. (f) X-ray sensitivity of the *p*-F-PEA_2_PbI_4_ single-crystal X-ray detector as a function of applied voltage. (g) The signal-to-noise ratio (SNR) of the *p*-F-PEA_2_PbI_4_ single-crystal device under different bias voltages and dose rates (1.06, 2.13, and 4.31 *μ*Gy_air_·s^−1^, respectively). (h) *p*-F-PEA_2_PbI_4_ single-crystal device responses to X-ray when turning the X-ray source on and off. The voltage bias is 5 V, and the dose rate is 5292 *μ*Gy_air_·s^−1^. (i) *p*-F-PEA_2_PbI_4_ single-crystal X-ray detector operating stability at 5292 *μ*Gy_air_·s^−1^ with ±5 V voltage bias, tested in ambient air without any encapsulation.

Many factors should be considered to fabricate good-performance X-ray detectors, such as the mechanical and atmospheric stability, absorption coefficient, detection area, etc. A large number of literatures have demonstrated the potential of perovskite single crystal materials for X-ray detectors previously, and the devices all show good performance [52–55]. In order to explore the specific effects of F substitution on the device performance, the corresponding X-ray detectors were prepared and tested. The inferior atmospheric stability of *p*-F-PEA_2_PbCl_4_ and the fragility of PEA_2_PbBr_4_ and *p*-F-PEA_2_PbBr_4_ single crystals make them difficult to meet either the working condition or thick film construction for X-ray detection. The X-ray attenuation ability can be determined by the absorption coefficient (*α*), which mainly depends on the atomic number (*Z*) and density (*ρ*), as shown in the equation: *α* = *ρ*Z^4^/AE^3^, where *A* is the atomic mass and *E* is the radiation energy. Heavy atomic constituents in PEA_2_PbI_4_ and *p*-F-PEA_2_PbI_4_ are beneficial for improving the X-ray absorption efficiency. The resistivity of *p*-F-PEA_2_PbI_4_ single crystal (6.33 × 10^13^  Ω cm) is greater than the corresponding value of PEA_2_PbI_4_ (1.42 × 10^13^  Ω cm), as shown in Figure S10. Moreover, the hole mobility was obtained from SCLC curve fitting by Mott-Gurney Law (Equation S1), and the value of *p*-F-PEA_2_PbI_4_ (28.11 cm^2^ V^−1^ S^−1^) was slightly larger than that of PEA_2_PbI_4_ (24.15 cm^2^ V^−1^ S^−1^). The measured photocurrent curves using the same illumination intensity, which was fitted by a modified Hecht equation (Equation S2) to obtain *μτ* product, are shown in Figure S11. The *p*-F-PEA_2_PbI_4_ single crystal shows a *μτ* product of 1.08 × 10^−4^ cm^2^ V^−1^, and the PEA_2_PbI_4_ single crystal gives a *μτ* product of 6.25 × 10^−5^ cm^2^ V^−1^. Based on the exhibited material properties, *p*-F-PEA_2_PbI_4_ is more suitable for X-ray detection than PEA_2_PbI_4_. The absorption spectra for the PEA_2_PbI_4_ and *p*-F-PEA_2_PbI_4_ and the semiconductor materials (Si and CdTe) in the photon energies ranging from 10 keV to 10 MeV are plotted in Figure 4(a), according to the photon cross-section database (NIST X-COM). The X-ray absorption coefficients of PEA_2_PbI_4_ and *p*-F-PEA_2_PbI_4_ are nearly the same, which are much higher than Si and slightly lower than CdTe. From 30 keV to 100 keV, the absorption of PEA_2_PbI_4_ and *p*-F-PEA_2_PbI_4_ is higher than 3D perovskite materials conventionally used in X-ray detection. When the thickness of the single crystal is larger than 1.25 mm, PEA_2_PbI_4_ and *p*-F-PEA_2_PbI_4_ can absorb more than 99% of X-ray photons, as shown in Figure 4(b). The as-prepared high-quality *p*-F-PEA_2_PbI_4_ single crystals prepared were integrated into direct horizontal X-ray detectors with Au/perovskite single-crystal/Au structure, as shown in Figure S12. The effective area of the X-ray detectors is 0.12 mm^2^. Figure 4(c) shows the *I-V* curve of the *p*-F-PEA_2_PbI_4_ single-crystal-based X-ray detector measured at different dose rates ranging from 50.364 *μ*Gy_air_·s^−1^ to 2094.3 *μ*Gy_air_·s^−1^. The *I-V* curves of the PEA_2_PbI_4_ single-crystal X-ray detector at the different dose rates ranging from 48.53 *μ*Gy_air_·s^−1^ to 396.4 *μ*Gy_air_·s^−1^ are shown in Figure S13. The *I-V* curves of the two devices are relatively smooth under different X-ray dose rates, which show a positive correlation with the applied X-ray dose rate. In addition, the lowest points in the curves of the PEA_2_PbI_4_ single-crystal-based X-ray detector gradually shift rightward, while the dose values increase, which is more obvious than that of the *p*-F-PEA_2_PbI_4_ counterparts. The ON/OFF current response of *p*-F-PEA_2_PbI_4_ single-crystal-based devices at 50 V bias voltage with the dose rate changing from 50.364 to 419.85 *μ*Gy_air_·s^–1^ is shown in Figure 4(d). The results prove that the device continues to switch at 50 V high voltage, maintaining good switching characteristics.

The ON/OFF current response of the PEA_2_PbI_4_ single-crystal X-ray detector is tested at 10 V, 20 V, 30 V, 40 V, and 50 V and dose rates from 48 *μ*Gy_air_·s^−1^ to 5.85 *μ*Gy_air_·s^−1^ to explore the device sensitivity (Figures 4(e) and S14(a)-S14(b)). The detection sensitivity, which can be extracted from the X-ray-generated current curve, is one of the critical parameters for the X-ray detector. Higher detection sensitivity is beneficial to low dose imaging and reduces radiation damage. The *p*-F-PEA_2_PbI_4_-based devices showed good performance, with the sensitivities measured to be 77.83 *μ*C·Gy_air_^−1^·cm^−2^ and 119.79 *μ*C·Gy_air_^−1^·cm^−2^ under the applied voltage as 10 V and 40 V, respectively. The sensitivity of the PEA_2_PbI_4_ single-crystal based X-ray detector increased from 37.12 *μ*C·Gy_air_^−1^·cm^−2^ to 114.74 *μ*C·Gy_air_^−1^·cm^−2^ with the applied voltage increased from 10 V to 40 V, as shown in Figure S14(c). Notably, the *p*-F-PEA_2_PbI_4_ single-crystal device exhibits better detection performance under low operating voltage. The detection limit is another important figure of merit for evaluating the performance of an X-ray detector for medical imaging and security screening. As illustrated in Figures 4(g) and S14(d), the detection limits of the *p*-F-PEA_2_PbI_4_ and PEA_2_PbI_4_ single-crystal X-ray detectors are as low as 1.06 *μ*Gy_air_·s^−1^ and 1.01 *μ*Gy_air_·s^−1^, at signal − to − noise (SNR) = 3, respectively [56–58]. The detection limits of the two detectors have reached the standards required by regular medical diagnostics (5.5 *μ*Gy_air_·s^−1^) [59, 60]. Figure 4(h) presents the radiation response of the *p*-F-PEA_2_PbI_4_ X-ray detector under 5 V bias voltage in ambient air. The on-state current *I_on_* is maintained at ~1.2 nA under 5292 *μ*Gy_air_·s^−1^, and the off-state current *I_off_* is at ~10.6 pA, resulting in a large ON/OFF ratio (~100) and a steady current under a fast switching test. The current of the *p*-F-PEA_2_PbI_4_ single-crystal-based X-ray detector remains stable at 5292 *μ*Gy_air_·s^−1^ with ±5 V bias voltage showing excellent working stability (Figure 4(i)). The performance of the *p*-F-PEA_2_PbI_4_ and PEA_2_PbI_4_ single-crystal X-ray detectors is almost the same in the detection sensitivity and detection limit tests. In addition, the performance of the *p*-F-PEA_2_PbI_4_ single-crystal X-ray detector shows no degradation under high-dose X-ray irradiation. *p*-F-PEA_2_PbI_4_ are much more stable under high-dose X-ray irradiation, resulting in better irradiation stability of the device, which satisfies the long-term working conditions of X-ray detectors. Both the fast switching test and the irradiation stability of the PEA_2_PbI_4_ single-crystal X-ray detector are inferior to the *p*-F-PEA_2_PbI_4_, as shown in Figure S15. The superior irradiation stability of *p*-F-PEA_2_PbI_4_ single crystal is attributed to F substitution, and the subsequence increased intermolecular forces. In general, *p*-F-PEA_2_PbI_4_ has the potential to be used in high-performance and high-stability X-ray detectors.

## 3. Discussion

In conclusion, we prepared a series of high-quality PEA_2_PbX_4_ and *p*-F-PEA_2_PbX_4_ single crystals through a simple solution method to study their properties changes after *p*-F substitution and halogen anion engineering. Through halogen engineering, the *π*-*π* stacking mode in the organic cation layer changes, which causes the crystal structure to change from the cubic (PEA_2_PbI_4_) or triclinic (PEA_2_PbCl_4_ and PEA_2_PbBr_4_) to monoclinic, which regulate the optical bandgap of the crystals. Besides, the *p*-F substitution enhances the overlap of electron clouds and thus enhances the internal diffusion effect of carriers as well as reduces the average lifetime of carriers, which is critical to improving device performance. In particular, *p*-F-PEA_2_PbI_4_ 2D perovskite single crystals with higher average atomic numbers have good X-ray detection performance, and its sensitivity can reach 119.79 *μ*C·Gy_air_^−1^·cm^−2^ when a voltage of 40 V is applied, which is higher that of PEA_2_PbI_4_. Furthermore, benefitting from the incorporation of F ion, the *p*-F-PEA_2_PbI_4_ single-crystal X-ray detector showed higher stability under high-dose X-ray irradiation, which could meet the long-term working requirement in the X-ray detector.

## 4. Materials and Methods

### 4.1. Materials

PEACl (99.5%), PEABr (99.5%), and PEACl (99.5%) were purchased from Xi'an Polymer Light Technology Corp. PbCl_2_ (99.999%), PbBr_2_ (99.9%), and PbI_2_ (99.9%) were purchased from Advanced Electron Technology Co., Ltd. *p*-F-PEACl (99.9%), *p*-F-PEABr (99.9%), and *p*-F-PEACl (99.9%) were provided by Teacher Gao Peng's research group. DMSO (≥99.7%) was purchased from J&K Scientific. DMF (≥99.9%) was purchased from Adamas-beta. GBL (≥ 99%) was purchased from Aladdin Reagent Ltd. CB (99.8%) was purchased from Sigma-Aldrich. The conductive silver paste is purchased from DJ912 of silver conductive paint of Mechanic. All the materials were used without purification.

### 4.2. Single-Crystal Preparation

1 M PEACl and 0.5 M PbCl_2_ were dissolved in 20 mL DMSO and stirred at 25 °C overnight to prepare the PEA_2_PbCl_4_ precursor solution. 1 M PEABr and 0.5 M PbBr_2_ were dissolved in 20 ml DMF and stirred at 25 °C overnight to prepare the PEA_2_PbBr_4_ precursor solution. 2 M PEAI and 1 M PbI_2_ were dissolved in 20 ml GBL and stirred at 80 °C overnight to prepare the PEA_2_PbI_4_ precursor solution. 1 M *p*-F-PEACl and 0.5 M PbCl_2_ were dissolved in 20 ml DMSO and stirred at 25 °C overnight to prepare the *p*-F-PEA_2_PbCl_4_ precursor solution. 1 M *p*-F-PEABr and 0.5 M PbBr_2_ were dissolved in 20 mL DMF and stirred at 25 °C overnight to prepare the *p*-F-PEA_2_PbBr_4_ precursor solution. 2 M *p*-F-PEAI and 1 M PbI_2_ were dissolved in 20 mL GBL and stirred at 80 °C overnight to prepare the *p*-F-PEA_2_PbI_4_ precursor solution. All precursor solutions were filtered using a 0.45 *μ*m filter before crystal growth. Single crystal of PEA_2_PbCl_4_, *p*-F-PEA_2_PbCl_4_, PEA_2_PbBr_4_, and *p*-F-PEA_2_PbBr_4_ was grown by slow solution evaporation. In the case of PEA_2_PbCl_4_, the filtered precursor solution kept at 30 °C. Several days later, 2D PEA_2_PbCl_4_ perovskite single crystals were obtained with a size of a few millimeters. Single crystal of PEA_2_PbI_4_ and *p*-F-PEA_2_PbI_4_ was crystallized at reduced temperature. In the case of PEA_2_PbI_4_, the filtered precursor solution was placed on the hot plate and kept at 80 °C for 24 h. Finally, the PEA_2_PbI_4_ single_-_crystal grew large constantly at a cooling rate of 2 °C per day. 2D *p*-F-PEA_2_PbI_4_ perovskite single crystals were also obtained by this method.

### 4.3. Characterization

XRD was performed with a Bruker D8 Discover X-ray diffractometer with a conventional Cu target X-ray tube set to 40 kV and 40 mA. PEA_2_PbX_4_ and *p*-F-PEA_2_PbX_4_ (X = Cl, Br, I) single-crystal XRD were performed with Bruker D8 Venture with Mo K*α* X-rays. Absorption spectra were measured using a Perkin-Elmer Lambda 950 UV-Vis-NIR spectrophotometer. Steady-state and time-resolved PL measurements of PEA_2_PbX_4_ and *p*-F-PEA_2_PbX4 (X = Cl, Br, I) were taken using an FLS980 Fluorescence Spectrofluorometer. All material characterizations were measured in the air without encapsulation. Crystallographic data have been deposited with Cambridge Crystallographic Data Centre (CCDC): Deposition number CCDC-2181443 for PEA_2_PbCl_4_, CCDC-2181444 for PEA_2_PbBr_4_, CCDC-2181438 for *p*-F-PEA_2_PbCl_4_, CCDC-2181439 for *p*-F-PEA_2_PbBr_4_, and CCDC-2181436 for *p*-F-PEA_2_PbI_4_. These structures can be obtained free of charge via https://www.ccdc.cam.ac.uk/structures/.

### 4.4. Device Fabrication

Hole-only devices were made by coating carbon on two opposite surfaces of the single crystals as electrodes. Electron-only devices were fabricated in the glovebox. First, 20 mg ml^−1^ PCBM CB solution was coated on one surface of a single crystal. Then the single crystal was annealed at 70 °C for 5 min. After annealing, Ag conductive paint was coated on single crystal surfaces covered with PCBM. The above operation is also performed on the other side of the single crystal. SCLC measurements were performed using a Keithley 4200 instrument.

### 4.5. Simulation Section

All calculations are carried out by using density functional theory based on the projector-augmented wave method implemented in the VASP code [61]. The exchange-correction functional is described by the Generalized Gradient Approximation with the Perdew-Burke-Ernzerhof functional [62].

### 4.6. Device Fabrication and Characterization

The PEA_2_PbI_4_ (5 × 5 × 2 mm^3^) and *p*-F-PEA_2_PbI_4_ (8 × 5 × 2 mm^3^) single crystals are used to make planar X-ray detectors. Au/Perovskite single-crystal/Au horizontal structure devices (Au is 100 nm, effective area of the interpolating device is 0.12 mm^2^) measure the X-ray response characterization. A tungsten anode X-ray tube (DX-DS2901/24) is used as the source. A Keysight B2902A source table provides the bias voltage and records the response current. The X-ray source operates at a constant voltage of 40 kV. The current was adjusted from 40 mA to 5 mA to tune the dose rate of the emitted X-rays. Several pieces of 2-mm-thick aluminum foils were inserted between the source and the CsPbBr_3_ single-crystal X-ray detector as attenuators. The X-ray dose rate is carefully measured using the Fluke Si diode (RaySafe X2 R/F) dosimeter. All X-ray response characterizations were performed directly in the dark air with optical and electrical shielding to reduce electromagnetic and ambient light interference. All measurements are performed at room temperature.

## Data Availability

Data used to support the findings of this study are included within the article and supplementary information file(s).

## Supplementary Materials

Supplementary Materials Figure S1. Schematic diagrams of the growth process with the slow solution evaporation method and reducing temperature method. Figure S2. Photograph of *p*-F-PEA_2_PbBr_4_single crystals under an optical microscope. Figure S3. Decomposition of PEA_2_PbCl_4_. Figure S4. Comparison of XRD patterns between PEA_2_PbCl_4_ and *p*-F-PEA_2_PbCl_4_ powder. Figure S5. UPS test results. Figure S6. PL spectrum of PEA_2_PbCl_4_ and *p*-F-PEA_2_PbCl_4_. Figure S7. Defect density of states in hole-only devices. Figure S8. Defect density of states in electron-only devices. Figure S9. Formation energies of halide ion vacancy defect for PEA_2_PbX_4_ and *p*-F-PEA_2_PbX_4_ Figure S10. The resistivity of PEA_2_PbI_4_ and *p*-F-PEA_2_PbI_4_ single crystal. Figure S11. Photoconductivity tests of corresponding PEA_2_PbI_4_ and *p*-F-PEA_2_PbI_4_ single crystal devices. Figure S12. Photograph of horizontal X-ray detectors (intercalating electrode) based on a *p*-F-PEA_2_PbI_4_ single crystal. Figure S13. I–V curves of the PEA_2_PbI_4_ single-crystal X-ray detector measured with dose rate from 48.53 to 396.4 *μ*Gy_air_·s^−1^. Figure S14. X-ray detector test results for PEA_2_PbI_4_. Figure S15. X-ray detector test results for PEA_2_PbI_4_ exposed to high X-ray dose rate. Table S1. X-ray diffraction data of PEA_2_PbX_4_ and *p*-F-PEA_2_PbX_4_ single crystal (X = Cl, Br, I). Table S2. Optical properties of PEA_2_PbX_4_ and *p*-F-PEA_2_PbX_4_ single-crystal samples. Table S3. Lifetime statistics for PEA_2_PbX_4_ and *p*-F-PEA_2_PbX_4_ single crystals. Table S4. The Bader charge transfer between supramolecular and Pb-X octahedron (Bader-1) and Bader charge transfer between supramolecular and supramolecular (Bader-2) of PEA_2_PbX_4_ and *p*-F-PEA_2_PbX_4_ (X = Cl, Br, I). Table S5. Trap densities of PEA_2_PbX_4_ and *p*-F-PEA_2_PbX_4_ single crystals. Table S6. DFT calculation of formation energies of halide ion vacancy defect for PEA_2_PbX_4_ and *p*-F-PEA_2_PbX_4_.
