# Supplementary material for: Low Trap Density Para-F Substituted 2D PEA2PbX4 (X = Cl, Br, I) Single Crystals with Tunable Optoelectrical Properties and High Sensitive X-Ray Detector Performance
Source: Research (Wash D C). 2022 Oct 10;2022:9768019. doi: 10.34133/2022/9768019 (PMC9590272; doi:10.34133/2022/9768019)
Supplement: Supplementary Materials — Figure S1. Schematic diagrams of the growth process with the slow solution evaporation method and reducing temperature method. Figure S2. Photograph of p-F-PEA2PbBr4single crystals under an optical microscope. Figure S3. Decomposition of PEA2PbCl4. Figure S4. Comparison of XRD patterns between PEA2PbCl4 and p-F-PEA2PbCl4 powder. Figure S5. UPS test results. Figure S6. PL spectrum of PEA2PbCl4 and p-F-PEA2PbCl4. Figure S7. Defect density of states in hole-only devices. Figure S8. Defect density of states in electron-only devices. Figure S9. Formation energies of halide ion vacancy defect for PEA2PbX4 and p-F-PEA2PbX4 Figure S10. The resistivity of PEA2PbI4 and p-F-PEA2PbI4 single crystal. Figure S11. Photoconductivity tests of corresponding PEA2PbI4 and p-F-PEA2PbI4 single crystal devices. Figure S12. Photograph of horizontal X-ray detectors (intercalating electrode) based on a p-F-PEA2PbI4 single crystal. Figure S13. I–V curves of the PEA2PbI4 single-crystal X-ray detector measured with dose rate from 48.53 to 396.4 μGyair·s−1. Figure S14. X-ray detector test results for PEA2PbI4. Figure S15. X-ray detector test results for PEA2PbI4 exposed to high X-ray dose rate. Table S1. X-ray diffraction data of PEA2PbX4 and p-F-PEA2PbX4 single crystal (X = Cl, Br, I). Table S2. Optical properties of PEA2PbX4 and p-F-PEA2PbX4 single-crystal samples. Table S3. Lifetime statistics for PEA2PbX4 and p-F-PEA2PbX4 single crystals. Table S4. The Bader charge transfer between supramolecular and Pb-X octahedron (Bader-1) and Bader charge transfer between supramolecular and supramolecular (Bader-2) of PEA2PbX4 and p-F-PEA2PbX4 (X = Cl, Br, I). Table S5. Trap densities of PEA2PbX4 and p-F-PEA2PbX4 single crystals. Table S6. DFT calculation of formation energies of halide ion vacancy defect for PEA2PbX4 and p-F-PEA2PbX4. [file 9768019.f1.docx]

**Supporting Material for**

**Low Trap Density Para-F Substituted 2D PEA_2_PbX_4_ (X = Cl, Br, I) Single-crystals with Tunable Optoelectrical Properties and High Sensitive X-Ray Detector Performance**

Jiayu Di^1^, Haojin Li^3^, Li Chen^1^, Siyu Zhang^1^, Yinhui Hu^1^, Kai Sun^1^, Bo Peng^1^, Jie Su^1^, Xue Zhao^1^, Yuqi Fan^4^, Zhenhua Lin^1^, Yue Hao^1^, Peng Gao^4*^, Kui Zhao^3*^, Jingjing Chang^1,2*^

^1^State Key Discipline Laboratory of Wide Band Gap Semiconductor Technology, School of Microelectronics, Xidian University, 710071, Xi'an, China.

^2^Advanced Interdisciplinary Research Center for Flexible Electronics, Academy of Advanced Interdisciplinary Research, Xidian University, 710071, Xi'an, China.

^3^Key Laboratory of Applied Surface and Colloid Chemistry, National Ministry of Education; Shaanxi Key Laboratory for Advanced Energy Devices; Shaanxi Engineering Lab for Advanced Energy Technology; Institute for Advanced Energy Materials; School of Materials Science and Engineering, Shaanxi Normal University, Xi’an 710119, China.

^4^CAS Key Laboratory of Design and Assembly of Functional Nanostructures, Fujian Institute of Research on the Structure of Matter, Chinese Academy of Sciences, Fuzhou 350002, China.
E-mail: jjingchang@xidian.edu.cn, Zhaok@snnu.edu.cn, peng.gao@fjirsm.ac.cn


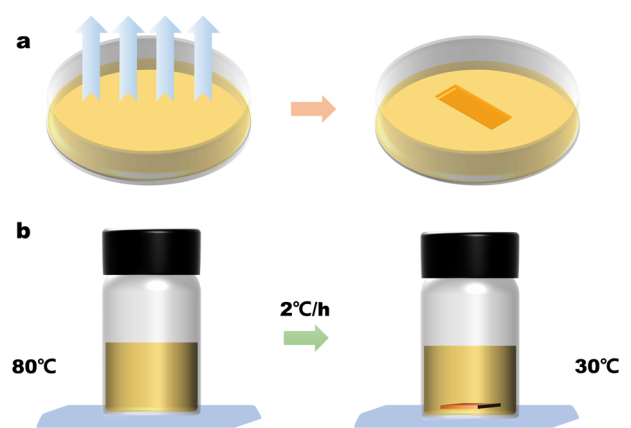


**Figure S1** (a) Schematic diagrams of the growth process with the slow solution evaporation method for PEA_2_PbCl_4_, *p*-F-PEA_2_PbCl_4_, PEA_2_PbBr_4_, and *p*-F-PEA_2_PbBr_4_ perovskite single crystals. (b) Schematic diagrams of the growth process with the reducing temperature method for PEA_2_PbI_4_ and *p*-F-PEA_2_PbI_4_ perovskite single crystals.


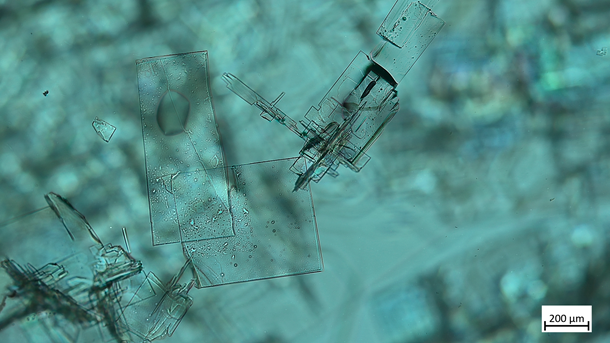


**Figure S2** Photograph of *p*-F-PEA_2_PbBr_4_ single crystals under an optical microscope.


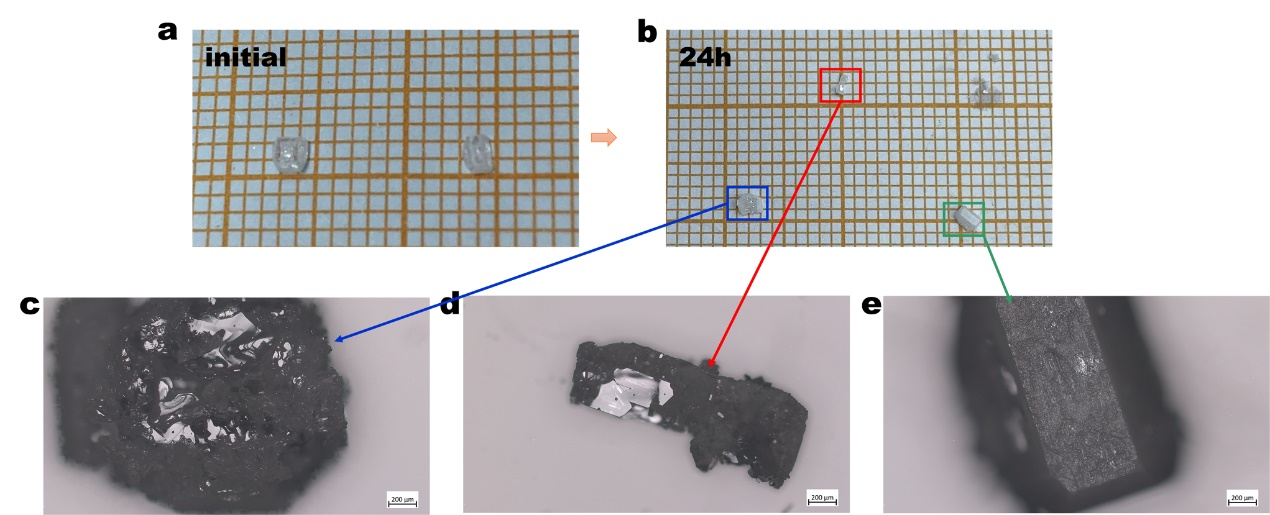


**Figure S3** (a) Photograph of *p*-F-PEA_2_PbCl_4_ single crystals (initial state: transparent). (b) Photograph of *p*-F-PEA_2_PbCl_4_ single crystals stored in air for 24h (current stage: chalk-like surface). (c) (d) and (e) Photographs of chalk-like surface crystals under an optical microscope. The surface became a non-reflective fuzzy surface composed of decomposed particles, and part of the uncorroded crystal (reflective surface) can be seen inside.

**Figure S4** Comparison of XRD patterns between PEA_2_PbCl_4_ and *p*-F-PEA_2_PbCl_4_ powder.

**Figure S5** (a) PL spectrum of PEA_2_PbCl_4_ and *p*-F-PEA_2_PbCl_4_.

**Figure S6** (a) The UPS spectrum of PEA_2_PbCl_4_. (b) The UPS spectrum of *p*-F-PEA_2_PbCl_4_. (c) The UPS spectrum of PEA_2_PbBr_4_. (d) The UPS spectrum of *p*-F-PEA_2_PbBr_4_. (e) The UPS spectrum of PEA_2_PbI_4_. (f) The UPS spectrum of *p*-F-PEA_2_PbI_4_.

**Figure S7** (a) Dark *I–V* plot of PEA_2_PbCl_4_ hole-only device using the space-charge-limited current (SCLC) method. (b) Dark *I–V* plot of the *p*-F-PEA_2_PbCl_4_ hole-only device using the SCLC method. (c) Dark *I–V* plot of PEA_2_PbBr_4_ hole-only device using the SCLC method. (d) Dark *I–V* plot of the *p*-F-PEA_2_PbBr_4_ hole-only device using the SCLC method. (e) Dark *I–V* plot of PEA_2_PbI_4_ hole-only device using the SCLC method. (f) Dark *I–V* plot of the *p*-F-PEA_2_PbI_4_ hole-only device using the SCLC method.

**Figure S8** (a) Dark *I–V* plot of PEA_2_PbCl_4_ electron-only device using the SCLC method. (b) Dark *I–V* plot of the *p*-F-PEA_2_PbCl_4_ electron-only device using the SCLC method. (c) Dark *I–V* plot of PEA_2_PbBr_4_ electron-only device using the SCLC. (d) Dark *I–V* plot of the *p*-F-PEA_2_PbBr_4_ electron-only device using the SCLC method. (e) Dark *I–V* plot of PEA_2_PbI_4_ electron-only device using the SCLC method. (f) Dark *I–V* plot of the *p*-F-PEA_2_PbI_4_ electron-only device using the SCLC method.

**Figure S9** Formation energies of halide ion vacancy defect for PEA_2_PbX_4_ and *p*-F-PEA_2_PbX_4_.

**Figure S10** The resistivity of PEA_2_PbI_4_ and *p*-F-PEA_2_PbI_4_ single crystal.


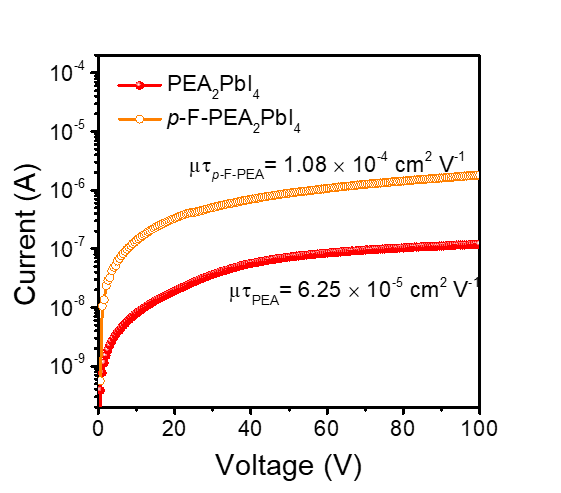


**Figure S11** Photoconductivity tests of corresponding PEA_2_PbI_4_ and *p*-F-PEA_2_PbI_4_ single crystal devices.


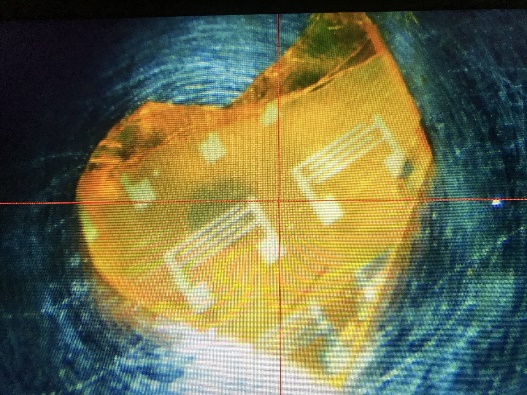


**Figure S12** Photograph of horizontal X-ray detectors (intercalating electrode) based on a *p*-F-PEA_2_PbI_4_ single crystal.

**Figure S13** *I – V* curves of the PEA_2_PbI_4_ single-crystal X-ray detector measured with dose rate from 48.53 to 396.4 μGy_air_·s^−1^.

**Figure S14** (a) ON/OFF current responses under various bias voltages (from 50 V to 10 V) and dose rates (from 48 to 5.85 μGy_air_·s^-1^) of the PEA_2_PbI_4_ single-crystal X-ray detector. (b) X-ray generated current density versus dose rate under different bias voltages. (c) X-ray sensitivity of the PEA_2_PbI_4_ single-crystal X-ray detector as a function of applied voltage. (d) The signal-to-noise ratio (SNR) of the PEA_2_PbI_4_ single-crystal device under different bias voltages and dose rates (1.01, 1.98, 3.99, and 6.09 μGy_air_·s^-1^, respectively).

**Figure S15** (a) PEA_2_PbI_4_ single-crystal device responses to X-ray when turning the X-ray source on and off. The voltage bias is 10 V, and the dose rate is 5.8 mGy_air_·s^-1^. (b) PEA_2_PbI_4_ single-crystal X-ray detector operating stability at 5.8 mGy_air_·s^-1^ with 10 V voltage bias, tested in ambient air without any encapsulation.

**Table S1** X-ray diffraction data of PEA_2_PbX_4_ and *p*-F-PEA_2_PbX_4_ single crystal (X = Cl, Br, I).

| **Chemical formula** | |  | ***p*-F-PEA_2_PbCl_4_** | ***p*-F-PEA_2_PbBr_4_** | ***p*-F-PEA_2_PbI_4_** | **PEA_2_PbCl_4_** | **PEA_2_PbBr_4_** | **PEA_2_PbI_4_**[1] |
| --- | --- | --- | --- | --- | --- | --- | --- | --- |
| Formula weight |  | 629.34 | | 807.16 | 995.14 | 593.36 | 771.20 | - |
| Temperature |  | 193 k | | 193k | 193k | 193k | 193k | - |
| Wavelength |  | 0.71073 | | 1.34139 | 0.71073 | 0.71073 | 0.71073 | - |
| Crystal system |  | Monoclinic | | Monoclinic | Monoclinic | Triclinic | Triclinic | - |
| Space group |  | P121/C1 | | C12/c1 | P21/C | P-1 | P-1 | C2/m |
| a |  | 16.9790(14) Å | | 33.174(5) Å | 16.5777(3) Å | 11.0642(3) Å | 11.5373(6) Å | 32.508(5) Å |
| b |  | 7.7467(7) Å | | 8.1042(12)Å | 8.5883(2) Å | 8.4546(3) Å | 11.5628(7) Å | 6.131(1) Å |
| c |  | 8.1041(7) Å | | 8.3805(13)Å | 8.7589(2) Å | 11.1854(5) Å | 17.3605(9) Å | 6.185(1) Å |
| α |  | 90º | | 90º | 90º | 80.780º | 99.623º | 90º |
| β |  | 97.738º | | 95.783º | 99.208º | 75.817º | 105.787º | 93.80(1)º |
| γ |  | 90º | | 90º | 90º | 89.996º | 90.020º | 90º |
| Volume |  | 1056.24(16) Å^3^ | | 2241.6(6)  Å^3^ | 1230.97(5) Å^3^ | 2064.77(10) Å^3^ | 2194.7(2) Å^3^ | 1230.000) Å^3^ |
| Z |  | 2 | | 4 | 2 | 4 | 2 | - |
| Density(calculated) |  | 1.979 g/cm^3^ | | 2.392 g/cm^3^ | 2.685 g/cm^3^ | 1.909 g/cm^3^ | 2.334g/cm^3^ | - |
| Absorption coefficient |  | 8.512 mm^-1^ | | 15.477 mm^-1^ | 11.887 mm^-1^ | 8.689 mm^-1^ | 14.970 mm^-1^ | - |
| Theta max |  | 27.499º | | 57.076º | 27.516º | 27.564º | 25.348º | - |
| Crystal size |  | 2× 2× 1mm^3^ | | 2× 2 × 1 mm^3^ | 4× 1 × 1 mm^3^ | 3× 3 × 0.5 mm^3^ | 3 × 3 × 2 mm^3^ | - |
| F000 |  | 600.0 | | 1488.0 | 888.0 | 1136.0 | 1424.0 | - |
| Reflection collected |  | 2410 | | 2217 | 2816 | 9179 | 7897 | - |
| Absorption correction |  | - | | 0.003-0.045 | 0.024-0.095 | 0.016-0.017 | 0.000-0.010 | - |
| Absorption correction type |  | Multi-scan | | Multi-scan | Multi-scan | Multi-scan | Multi-scan | - |

**Table S2** Optical properties of PEA_2_PbX_4_ and *p*-F-PEA_2_PbX_4_ single-crystal samples.

| **Materials** | **λ_light_**  **[nm]** | **E_g_**  **[eV]** | **PL peak**  **[nm]** |
| --- | --- | --- | --- |
| PEA_2_PbCl_4_ | 335nm | 3.46 | 521 |
| *p*-F-PEA_2_PbCl_4_ | 335nm | 3.42 | 528 |
| PEA_2_PbBr_4_ | 365nm | 2.88 | 412 |
| *p*-F-PEA_2_PbBr_4_ | 365nm | 2.81 | 419 |
| PEA_2_PbI_4_ | 375nm | 2.26 | 524 |
| *p*-F-PEA_2_PbI_4_ | 375nm | 2.24 | 525 |

**Table S3** Lifetime statistics for PEA_2_PbX_4_ and *p*-F-PEA_2_PbX_4_ single crystals.

| **Materials** | **A_1_** | **t_1_** | **A_2_** | **t_2_** | **t_av_** |
| --- | --- | --- | --- | --- | --- |
| PEA_2_PbCl_4_ | 37.0% | 8.52ns | 63.0% | 1.06ns | 3.82ns |
| *p*-F-PEA_2_PbCl_4_ | 42.1% | 3.60ns | 57.9% | 0.60ns | 1.87ns |
| PEA_2_PbBr_4_ | 28.2% | 2.63ns | 71.8% | 11.20ns | 8.79ns |
| *p*-F-PEA_2_PbBr_4_ | 59.8% | 6.51ns | 40.2% | 1.89ns | 4.65ns |
| PEA_2_PbI_4_ | 77.9% | 0.62ns | 22.1% | 3.34ns | 1.22ns |
| *p*-F-PEA_2_PbI_4_ | 24.1% | 1.29ns | 75.9% | 0.26ns | 0.51ns |

**Table S4** The Bader charge transfer between supramolecular and Pb-X octahedron (Bader-1) and Bader charge transfer between supramolecular and supramolecular (Bader-2) of PEA_2_PbX_4_ and *p*-F-PEA_2_PbX_4_ (X = Cl, Br, I).

| **Structure** | **PEA_2_PbI_4_** | **PEA_2_PbBr_4_** | **PEA_2_PbCl_4_** |
| --- | --- | --- | --- |
| Bader-1 (eV) | 5.33 | 5.78 | 6.08 |
| Bader-2 (eV) | 0.88 | 1.01 | 1.29 |
| **Structure** | ***p*-F-PEA_2_PbI_4_** | ***p*-F-PEA_2_PbBr_4_** | ***p*-F-PEA_2_PbCl_4_** |
| Bader-1 (eV) | 5.18 | 5.39 | 5.62 |
| Bader-2 (eV) | 1.21 | 1.46 | 1.98 |

**Table S5** Trap densities of PEA_2_PbX_4_ and *p*-F-PEA_2_PbX_4_ single crystals.

| **Materials** | **PEA_2_PbCl_4_** | ***p*-F-PEA_2_PbCl_4_** | **PEA_2_PbBr_4_** | ***p*-F-PEA_2_PbBr_4_** | **PEA_2_PbI_4_** | ***p*-F-PEA_2_PbI_4_** |
| --- | --- | --- | --- | --- | --- | --- |
| Electron trap density  (cm^-3^) | 4.16×10^10^ | 3.67×10^9^ | 2.11×10^10^ | 1.08×10^10^ | 1.47×10^10^ | 5.53×10^9^ |
| Hole trap density  (cm^-3^) | 1.56×10^11^ | 2.39×10^10^ | 1.83×10^11^ | 8.99×10^10^ | 1.34×10^11^ | 1.73×10^10^ |

**Table S6** DFT calculation of formation energies of halide ion vacancy defect for PEA_2_PbX_4_ and *p*-F-PEA_2_PbX_4_

| **Structure** | **Formation energy (eV)** | **Structure** | **Formation energy** |
| --- | --- | --- | --- |
| PEA_2_PbCl_4_ | 5.3163 | *p*-F-PEA_2_PbCl_4_ | 5.9588 |
| PEA_2_PbBr_4_ | 5.0563 | *p*-F-PEA_2_PbBr_4_ | 5.5235 |
| PEA_2_PbI_4_ | 3.9894 | *p*-F-PEA_2_PbCl_4_ | 5.0125 |

**Equation S1** Mott–Gurney Law[2]–[4]

$J=\frac{9}{8}\text{ε}_{0}\text{ε}_{\text{r}}\text{μ}\frac{V^{2}}{\text{d}^{3}}$

where *J* is the current density, *V* is the applied voltage, ε_r_ is the relative dielectric constant, ε_0_ is the vacuum permittivity, μ is the mobility of single crystals, and d is the thickness of materials.

**Equation S2** Modified Hecht equation[5]-[7]

$I\text{=}\frac{\text{I}_{0}\text{μτ}V}{\text{L}^{2}}\frac{\text{1-exp(-}\frac{\text{L}^{2}}{\text{μτ}V}\text{)}}{\text{1+}\frac{\text{Ls}}{V\text{μ}}}$

where I_0_ is the saturated photocurrent, L the thickness of materials, τ is the lifetime of the material, and *V* the applied bias.

[1] D. Ma, Y. Fu, L. Dang et al, “Single-crystal microplates of two-dimensional organic–inorganic lead halide layered perovskites for optoelectronics,” *Nano Res.*, vol. 10, no. 6, pp. 2117–2129, 2017, doi: 10.1007/s12274-016-1401-6.

[2] D. Ju, X. Jiang, H. Xiao, X. Chen, X. Hu, and X. Tao, “Narrow band gap and high mobility of lead-free perovskite single crystal Sn-doped MA 3 Sb 2 I 9,” *J. Mater. Chem. A*, vol. 6, no. 42, pp. 20753–20759, 2018, doi: 10.1039/C8TA08315K.

[3] Z. Xu *et al.*, “Exploring Lead-Free Hybrid Double Perovskite Crystals of (BA)2CsAgBiBr7 with Large Mobility-Lifetime Product toward X-Ray Detection,” *Angew. Chemie - Int. Ed.*, vol. 58, no. 44, pp. 15757–15761, 2019, doi: 10.1002/anie.201909815.

[4] Y. Liu *et al.*, “20-mm-Large Single-Crystalline Formamidinium-Perovskite Wafer for Mass Production of Integrated Photodetectors,” *Adv. Opt. Mater.*, vol. 4, no. 11, pp. 1829–1837, 2016, doi: 10.1002/adom.201600327.

[5] C. C. Stoumpos *et al.*, “Crystal growth of the perovskite semiconductor CsPbBr3: A new material for high-energy radiation detection,” *Cryst. Growth Des.*, vol. 13, no. 7, pp. 2722–2727, 2013, doi: 10.1021/cg400645t.

[6] Y. Liu *et al.*, “Surface-Tension-Controlled Crystallization for High-Quality 2D Perovskite Single Crystals for Ultrahigh Photodetection,” *Matter*, vol. 1, no. 2, pp. 465–480, Aug. 2019, doi: 10.1016/j.matt.2019.04.002.

[7] H. Li *et al.*, “Sensitive and Stable 2D Perovskite Single-Crystal X-ray Detectors Enabled by a Supramolecular Anchor,” *Adv. Mater.*, vol. 32, no. 40, pp. 1–9, 2020, doi: 10.1002/adma.202003790.
